# Integrating temperature-dependent life table data into Insect Life Cycle Model for predicting the potential distribution of *Scapsipedus icipe* Hugel & Tanga

**DOI:** 10.1371/journal.pone.0222941

**Published:** 2019-09-25

**Authors:** Magara H. J. Otieno, Monica A. Ayieko, Saliou Niassy, Daisy Salifu, Azrag G. A. Abdelmutalab, Khamis M. Fathiya, Sevgan Subramanian, Komi K. M. Fiaboe, Nana Roos, Sunday Ekesi, Chrysantus M. Tanga

**Affiliations:** 1 School of Agriculture and Food Security, Jaramogi Oginga Odinga University Science and Technology (JOOUST), Bondo, Kenya; 2 International Centre of Insect Physiology and Ecology (icipe), Nairobi, Kenya; 3 The International Institute of Tropical Agriculture (IITA), B.P. 2008 (Messa), Nkolbisson, Yaoundé, Cameroon; 4 University of Copenhagen, Department of Nutrition, Exercise and Sports, Rolighedsvej, Frederiksberg C, Denmark; Natural Resources Canada, CANADA

## Abstract

*Scapsipedus icipe* Hugel and Tanga (Orthoptera: Gryllidae) is a newly described edible cricket species. Although, there is substantial interest in mass production of *S*. *icipe* for human food and animal feed, no information exists on the impact of temperature on their bionomics. Temperature-dependent development, survival, reproductive and life table parameters of *S*. *icipe* was generated and integrated into advanced Insect Life Cycle Modeling software to describe relative *S*. *icipe* population increase and spatial spread based on nine constant temperature conditions. We examined model predictions and implications for *S*. *icipe* potential distribution in Africa under current and future climate. These regions where entomophagy is widely practiced have distinctly different climates. Our results showed that *S*. *icipe* eggs were unable to hatch at 10 and 40°C, while emerged nymphs failed to complete development at 15°C. The developmental time of *S*. *icipe* was observed to decrease with increased in temperature. The lowest developmental threshold temperatures estimated using linear regressions was 14.3, 12.67 and 19.12°C and the thermal constants for development were 185.2, 1111.1- and 40.7-degree days (DD) for egg, nymph and pre-adult stages, respectively. The highest total fecundity (3416 individuals/female/generation), intrinsic rate of natural increase (0.075 days), net reproductive rate (1330.8 female/female/generation) and shortest doubling time (9.2 days) was recorded at 30°C. The regions predicted to be suitable by the model suggest that *S*. *icipe* is tolerant to a wider range of climatic conditions. Our findings provide for the first-time important information on the impact of temperature on the biology, establishment and spread of *S*. *icipe* across the Africa continent. The prospect of edible *S*. *icipe* production to become a new sector in food and feed industry is discussed.

## Introduction

Insect consumption is a practice that has increased but is still restricted to a few countries. Nearly 2000 species are reported edible and approximately 300 million people consume insects [1]. In Africa, nearly 500 consumable insects are reported to be eaten by human beings [2]. Edible insects form part of the diet of many Africans particularly as a delicacy. However, edible insect supply largely depends on wild harvesting. With the increasing advocacy of economically feasible and environmental sustainability, the blueprint of insect mass production has become a salient target [3].

In Sub-Saharan Africa countries, many cricket species are considered for large-scale production [4–6]. These include *Acheta domesticus* Linnaeus, *Gryllus bimaculatus* De Geer and *Gryllus texensis* [3, 5–7]. Furthermore, a newly described indigenous cricket *Scapsipedus icipe* Hugel and Tanga was recently reported as one of the most promising species for use as food and feed in the continent [8–9]. In Kenya, *S*. *icipe* is the dominant and most wide-spread species across the low, mid and highlands [8].

Commercial insect farming systems are emerging globally due the urgent need for both viable and sustainable alternative protein sources for improving the productivity of livestock and aquaculture sectors [10]. Recent scientific advances have demonstrated the potential of insects as an alternative sustainable protein-rich ingredient to the expensive conventional protein sources (i.e., soybeans, fish oil, fishmeal, seed cakes and several other grains), which accounts for 60–70% of production costs [11]. The animal feed sector in East Africa is organized, regulated and has broadly accepted insects as an alternative protein source [12]. Furthermore, the production of insects for human consumption to address acute food shortages and malnutrition is widely being promoted world [3, 10–11, 13].

Although *S*. *icipe* is consumed as food in Africa, information on their rate of development, thermal thresholds, survival, fecundity, fertility and longevity are lacking. Temperature is regarded as one of the most significant environmental conditions that impacts insect evolution, adult lifespan, survival, sex ratio, fertility and distribution [14–18]. Among the above listed performance indicators, the rate of development is conventionally used to quantify the effect of temperature. Preceding studies have shown that each insect population has an optimal temperature at which its development is most favorable as well as lower and upper temperature limits beyond which they cannot develop [18–23]. Several studies have demonstrated that stage-specific mortality carried out within widely varying temperatures are valuable tools in the study of population dynamics of insects as they help to measure the rate of mortality at different stages in the life cycle and determine optimal, lower and upper limits of developmental thresholds [15, 17–18].

Based on these abiotic factors, distinct models have been employed to estimate the correlation between these variables especially temperature and development processes of insects. Generally, the interrelations between temperature and process rates are more or less linear at moderate temperatures and curvilineal at maximum points [24]. Linear models have many times been applied to determine the lower development temperature limit and thermic constant within a narrow temperature range, with an assumption that the upper development temperature limit is beyond the linear part of the correlation [19, 25]. Nonlinear models have been advanced to relate the whole relationship that exists between the development of an insect species and its developmental temperature, which ranges from the lower developmental temperature threshold to an upper temperature threshold [23, 25–28]. Therefore, details on the thermic requirements of a newly described insect species’ development would have significant implications for mass production programs, as temperature determines the increase of the population growth and size of any insect species and their differences under varying conditions [17–18, 29].

Considering the prospects of *S*. *icipe* in addressing food security, nutrition and employment in the region [9], it is crucial to carry out comprehensive studies to understand the influence of different temperature regimes on the bionomics of *S*. *icipe*, which is a prerequisite for mass production and quality control programs, if the optimal conditions are known. Based on these findings, we also wanted to know the potential distributional range of this new edible insect species in Africa using climate suitability map. The climate suitability map highlights areas with lower, optimal and upper developmental temperature thresholds where the species can survive and develop, thus providing enabling environment to inform policy decision making with regards to conservation of this new species where ever they may occur. Thus, temperature-dependent population growth life-cycle simulation models were used to assess their spread in various agro-ecological zones in Africa. The findings would inform geographically-targeted policies in order to guide biodiversity hotspots for the conservation of *S*. *icipe*, an important, abundant, and often ignored component of biodiversity. The strong performance of the model for hotspot prediction emphasizes the importance of including new species’ natural history information [9] for conservation decision-making, which could be easily adaptable to other insect species [30]. The climate change impacts on *S*. *icipe* potential distribution and conservation is also discussed.

## Materials and methods

### Insect colony

The colony of *S*. *icipe* was initiated from wild populations [425 nymphs and 366 adult insects (248 females and 118 males)] trapped from the grassland fields in Coast, Central and Western of Kenya during a country-wide survey conducted in 2016. The wild field populations were reared according to the methods described by Magara et al. [9]. Adult crickets and nymphs were fed on formulated diets, which consisted of a mixture of different feedstocks (cornmeal, wheat bran, pumpkin leaves meal, fish offal meal and soybean waste meal) and maintained at 30°C; 80±5% relative humidity and photoperiod of L12:D12, following the protocol described by Melisa [31] with slight modifications. The crickets were provided water regularly on soaked cotton balls (5 cm diameter) [9]. The stock colony was refreshed twice each year by introducing the F1 generation of newly harvested wild population of *S*. *icipe* to minimize inbreeding. The stock was raised for over eight generations before the commencement of the experiments. The stock colony is maintained at the International Centre of insect physiology and ecology (*icipe*), Duduville campus, Nairobi (1.221S, 36.896E; 1616 m above sea level) in the Animal Rearing and Containment Unit (ARCU). The cricket populations used in the present where obtained from the ARQU.

### Development, survival, growth and reproduction monitoring experimental set up

The thermal effect on the development, survivorship/mortality and reproduction was investigated in a thermostatically controlled incubators (Sanyo, MIR- 554) at nine constant temperature regimes (10, 15, 20, 23, 25, 30, 35, 37 and 40°C), 80 ± 5% r.h. and L12:D12. A portable digital thermo-hygrometer was placed inside each incubator to record the temperature and relative humidity.

### Thermal effect on development time, adult longevity, lifespan and stage-specific mortality of *Scapsipedus icipe*

To collect eggs of same age, 500 adult females and 500 adult males of *S*. *icipe* were placed in transparent plastic rearing cages (60 x 50 x 60 cm) with an opening sealed with a nylon mesh net to offer adequate ventilation [8–9, 32–33]. The crickets were fed on an artificial feed as described above [9, 31]. Moist cotton balls were provided in each cage to serve as oviposition medium. Freshly laid eggs (2–3 hours old) were carefully detached from the cotton balls using a fine camel hair brush. The eggs collected were incubated individually on thinly spread moist cotton wool, which was placed in a well-ventilated plastic cage (16 × 7 × 9 cm). Each temperature treatment had a total of 100 eggs, each serving as a replicate. For each temperature regime, each egg was monitored thrice daily (every six hours) for hatchability and percent mortality. The emerged nymphs were supplied with 1 mg of the artificial diet and water as described above. The duration of each moult (developmental time) and survivorship was recorded daily until adult emergence for each temperature treatment. The sex ratio of adults emerged from each temperature regime was also recorded. The survivorship data for each life stage was recorded and used to calculate stage-specific survivorship and mortality for each nymphal developmental period.

### Thermal effect on body length and body weight of adult *Scapsipedus icipe*

To evaluate the impact of temperature on the body weight and body length of adult male and female *S*. *icipe*, 10 adult males and 10 adult females were randomly selected from those that emerged as adult in the above experiment. The crickets were transferred individually into transparent plastic cages (15 cm length x 10 cm wide 6cm depth) secured with a nylon screen. Water was provided *ad libitum* in Petri dishes using soaked pumice granules. Crickets were fed for 7 days until sexual maturity, when all the body colourations were attained. Thereafter, the weight of each cricket was measured using a digital electronic weighing balance with a precision of 0.0001 g (Kern and Sohn, D-72336 Ballngen, Germany). The width of each cricket’s body was cautiously measured using digitalized Vernier callipers [34]. For the length of randomly selected individual crickets, each was placed in a thin transparent plastic container with a diameter of 8 cm and height of 12 cm. The container was then gently placed on a well-calibrated ruler, essential for traceability and accuracy measurement of the length of the cricket. Thereafter, a smaller transparent container with 4 cm diameter and 6 cm height was carefully used to cover the cricket inside the bigger container to significantly reduce mobility. Once stable the measurement of the length of the cricket was recorded with precision.

### Thermal effect on female fecundity

Adult crickets emerged (2–3 hours old) from the previous experiment were randomly selected, paired and kept separately in aerated transparent cages (20 x 20 x 15 cm) subjected to the different temperature regimes. Each pair of crickets were provided water on pumice granules in a Petri dish and fed similar diets as described above. An oviposition substrate that consisted of a soaked cotton ball was placed inside sterile Petri dish (60 x 15 mm) in each cage for egg laying and to maintain the relative humidity. The oviposition substrate was exposed for 24 hours in each cage and thereafter eggs laid were counted daily using a moistened fine camel’s hair brush. Total number of freshly laid eggs per day per female throughout the lifespan were collected and recorded within a uniform time interval of 2–3 hrs. The rate of hatchability of eggs produced at each temperature treatment was carefully established. Additional, parameters measured included: adult pre oviposition period (APOP; the timeframe between the emerged adult female and the beginning of egg laying), the total pre oviposition period (TPOP; the duration from newly hatched pinhead crickets to the time of first egg laying), the oviposition duration (refers to the entire egg laying period of the cricket), the day by day fecundity and total fecundity (sum number of eggs laid by an individual during its reproductive period), Post oviposition period (POP; Period between last egg laid and death of cricket), survival and longevity of each adult in each treatment temperature were recorded. A total of 20 pairs of adult crickets were observed at each tested temperature regime.

An additional cohort of emerged adult (50 female and 50 male) crickets (2–3 hours old) from each temperature treatment was kept separately in groups in transparent Perspex cages (45 x 45 x 50 cm). From each of the different temperature treatments, the longevity and survival rate of male and female crickets were recorded separately.

### Statistical analyses and modelling

The data on the developmental time of the various life stages of *S*. *icipe*, adult longevity and females’ fecundity were compared across temperatures using one-way analysis of variance (ANOVA; P < 0.001) and the means were separated using Student-Newman-Keul’s test at a significance of 0.05 (P < 0.05) in R Statistic (Version 3.3.3) [35]. Temperature-dependent models or functions were used to model the demographic characteristics of *S*. *icipe*, by employing the Insect Life Cycle Modelling program (ILCYM, version 3.0) [36]. This program has a function builder that fits non-linear models as descriptors of correlation between temperature and insect development characteristics. The function builder then utilizes its inbuilt statistical criteria, combined with the data on the biology of the insect species under investigation, selects the best fitting functions to describe the intermediate temperature-driven processes. After that, it amasses the processes into a phenological model for the population under assessment [36]. Akaike’s Information Criterion (AIC), which defines the best fit of an estimated statistical model and the coefficient of determination R^2^, which is the portion of variation explained by the function, as statistics used for model selection. The lower the AIC, the better the model while the higher the R^2^, the better the model. A female ratio of 0.5 was taken into account for all the temperature treatments under study.

### Thermal effect on the development rate of immature stages of *Scapsipedus icipe*

The rate of development of juveniles of *S*. *icipe* was evaluated as an inverse of development time (rate of development = 1/median of developmental time) [37], for each juvenile stage and the total developmental duration from egg to adult stage, and then plotted versus temperature. The linear part of the rate of the developmental curve was modelled employing linear regression analysis hence establishing the relationship between the rate of development and temperature [36]. The following linear equation was adopted:

r(T)=a+bT (1)

whereby r(T) refers to the rate of development at temperature T, and constants a and b are estimated values of the intercept and slope, accordingly. The lower limit temperature (T_min_) was determined at the intersection of the regression line at r (T) = 0, $T_{min}=-\frac{a}{b}$. Thermal constant, K also referred to as degree-day (DD) requirements) were evaluated using the inverse of the slope of the inserted linear regression line [38].

The linear model cannot correctly capture the rate of development at extreme temperatures. Therefore, by using AIC selection criteria [39] and R^2^, a nonlinear Logan 1 model [26] and Allahyari model [23] were fitted between the rate of development, r (T) and temperature, T to determine the correlation between temperature and development by use of the Marquardt algorithm equation [40]. The Logan 1 model was applied to the egg stage while Allahyari model was applied to the young nymphal stages (i.e., nymph and pre-adult data generated).

The Logan 1 equation is provided as:

$$r\;(T)=Y\;\left(\exp\;\left(p.T\right)-\exp\;\left(p.T_{max}-\frac{T_{max}-T}{v}\right)\right)$$ (2)

whereby r (T) refers to the development rate of the insect at temperature T; while Y, p and v are constants values. This model estimated the upper lethal temperature where there is no measurable development and lower temperature limit below which the linear regression estimated no measurable development.

The Allahyari equation is given as:

$$r\;(T)=P.{\left(\left(\frac{T-T_{min}}{T_{max}-T_{min}}\right)\right)}^{n}).(1-{\left(\frac{T-T_{min}}{T_{max}-T_{min}}\right)}^{m}$$ (3)

whereby r (T) refers to the development rate at temperature T, P refers to the number of model parameters while n and m are constants values, T_max_ is the maximum lethal temperature and T_min_ is the minimum lethal temperature [23]. The maximum lethal temperature (T_max_) and the temperature for the shortest development time was estimated from the Logan models for each immature stage and the total development from egg to adult. Optimum temperature (T_opt_) for survival (= temperature for lowest mortality) was estimated from the models for each immature stage.

### Thermal effect on the mortality rate of immature stages models

Mortality of eggs and nymphs at various constant temperatures were best determined by the Wang 2 equation [41] whereas Wang 3 equation [41] provided the best curve for mortality of the pre-adult, respectively. The Wang 2 and Wang 3 equations were given as:

$$m\;(T)=1-\frac{1}{e^{\left((1+e(-\frac{T-T_{l}}{B}))(1+e(-\frac{T_{h-T}}{B}))x\;H\right)}}$$ (4)

$$m\;(T)=1-\frac{1}{e^{\left((1+e(-\frac{T-T_{opt}}{B}))(1+e(-\frac{T_{opt-T}}{B}))x\;H\right)}}$$ (5)

whereby m (T) is the mortality rate at temperature T (°C), T_l_ is the minimum lethal temperature, T_h_ is maximum lethal temperature, T_opt_ is the optimal temperature for survival, while B and H are constant values of model parameters. In both Wang 2 and Wang 3 models refers to the natural exponential.

### Temperature- dependent reproduction models

A Wang 7 function [41] was considered as a suitable model in assessing the impacts of temperature on fecundity. At the same time, the relative oviposition frequency, which demonstrates the portion of total lifespan reproductive capacity that passes in each time period, was assessed in connection to the normalized age of adult female crickets at a particular temperature. The cumulative oviposition rate was graphed against the normalized age indicated as a ratio of age in days over the average survival time. The Gamma equation [42] was utilized to fit the experimental datasets and further help to evaluate parameters, for example, H, B1 and B_h_. The Wang 7 function was provided as:

$$f\;(T)=\frac{\frac{1-H}{\left(exp\;(1+exp\;(-(T-T_{opt}\right)}}{B_{1}}.\frac{\left(1+exp\;(-(T_{opt}-T\right)}{B_{h}}$$ (6)

whereby f (T) refers to the fecundity at temperature T (°C), x is the maximum fecundity, T_opt_ is the temperature at which maximum fecundity occurs, and H, B1 and *B*_*h*_ are other model parameters.

An exponential function was applied to recount the age-specific fecundity rate per temperature. The cumulative rate of oviposition was then plotted against normalized females’ age presented as a ratio of the age of female crickets in days divided by average survival time. The exponential equation function is given as:

$$O(E)=1-e^{-(aE+BE^{2}+cE^{3})}$$ (7)

whereby O(E) refers to the cumulative oviposition frequency female crickets, E is the normalized age of female crickets indicated as a ratio of the age of female cricket in days and average survival time while a, b and c are function parameters.

### Thermal effect on adult lifespan and senescence models

The cumulative frequencies of the adult crickets’ life span and treatment temperatures were plotted versus normalized developmental times by fitting in Hilbert and Logan 3 [43] function for adult female crickets and Exponential simple function for adult male crickets.

Hilbert and Logan 3 function is given as:

$$s\;(T)=trid\;\left(\frac{(T-T_{min})²}{(T-T_{min})²+D}-e^{\left(-(T_{\max-\frac{\left(T-T_{min}\right)}{D_{t}}}\right)}\right)$$ (8)

whereby T is temperature, T_max_ is the thermal maximum, T_min_ refers to the lowest thermal limit below which development cannot take place (= temperature development threshold) and T_max_, is the uppermost thermal limit beyond which development will not take place while Dt, D, and trid are parameters to be estimated by least squares nonlinear analysis. The least squares estimated value of T_max_ was used as a limit mark for viable temperature conditions for *S*. *icipe*.

Exponential Simple function was given as:

$$s\;(T)=b1*e^{b2*T}$$ (9)

whereby s (T) is the rate of adult senescence while b1 and b2 are function parameters.

The oviposition was modelled by looking at the three temperature-dependent functions: thus, temperature dependent total fecundity, age-related oviposition frequency and age-specific survival of adult crickets. Hilbert and Logan 3 formulae were applied to evaluate the effects of various constant treatment temperatures on a total number of eggs oviposited by a female cricket throughout her lifetime.

### Life table parameters for *Scapsipedus icipe* simulation

The models developed for *S*. *icipe* demographic characteristics were compiled and utilized to determine the life table parameters. Using stochastic simulation tool [44] in ILCYM, the life table parameters such as the gross reproductive rate (GRR), which refers to the mean number of nymph daughters bred by a living female during her reproductive period, the net reproductive rate (R_o_), which also takes into account mother mortality, the intrinsic rate of increase (r_m_), which is summarized from all the demographic variables indicating the ability of a population to grow under particular environmental conditions, the mean generation time (T), and doubling time (Dt) for the population were estimated. During simulations, 100 individuals at the egg stage were involved and the simulation was conducted for six constant temperatures from 20, 23, 25, 30, 35 and 37°C. The simulation was replicated six times for each temperature. This offered to signify how the change in temperature can influence *S*. *icipe* population growth and to estimate the temperature thresholds of *S*. *icipe* population growth. The simulated life table parameters were analysed using ANOVA in R (Version 3.3.0) [35].

### Current spatial simulations and mapping of *Scapsipedus icipe* in Africa

The temperature information used for spatial simulations in the current scenario was obtained from WorldClim at http://www.worldclim.org. The information was a set of worldwide climate layers (grids) with completely different spatial resolutions that contain monthly average minimum, maximum and mean temperatures that were interpolated from historical temperature records worldwide (NOAA data) between 1950 and 2000. The temperature data is well recorded in Hijmans et al. [45]. For spatial population simulations and model output validations at totally different locations (point-by-point) temperature information directly obtained from native weather stations were used.

### Potential African wide establishment of *Scapsipedus icipe*

For simulating population parameters for *S*. *icipe* for the year 2050 (future scenario) we employed downsized data of the SRES-A1B [46] to project temperature changes. The predictions developed on the WorlClim data bank are reported by Govindasamy et al [47]. The downsizing of data which was conducted by Ramirez and Jarvis [48] is freely available at http://gisweb.ciat.cgiar.org/GCMPage. For point-by-point analysis, empirical data were chosen as baseline data, and we assumed increases of 1, 1.6, 2.4 and 2.8°C global temperature that may be the average global temperature change according to the A1B model within the years 2030, 2050, 2080, and 2100, respectively.

## Results

### Effect of temperature on stage development, sex ratio, adult longevity and lifespan of *Scapsipedus icipe*

The eggs of *S*. *icipe* were unable to hatch at 10 and 40°C, while emerged nymphs failed to complete development to adult at 15°C. The nymphal stages of *S*. *icipe* were found to complete development within the temperature range of 20–37°C. The number of moults from 1^st^ nymphal instar to preadult stage was observed to decrease significantly with increasing temperature. The highest number of moults were recorded at 20°C (10 moults), followed by 23°C (9^th^ moults) and 25–37°C (8^th^ moults) (Table 1).

**Table 1:** Mean (±SE) developmental time (days) of different life stages of *Scapsipedus icipe* at different constant temperatures.

Life stage	Temperature (°C)								
	10*	15	20	23	25	30	35	37	40*
Egg incubation period	^†^	76.20±0.05 a	42.60±0.05 b	31.03±0.09 c	20.20±0.08d	10.40±0.05 e	9.61±0.05 e	9.80±0.08 e	^†^
1^st^ nymphal duration	^†^	27.20±0.35a	14.72±0.19b	13.06±0.14b	9.35±0.06c	5.27±0.04d	5.46±0.05d	5.41±0.05d	^†^
2^nd^ nymphal duration	^†^	29.86±0.21a	15.22±0.18b	14.04±0.14b	9.63±0.06c	5.65±0.06d	5.88±0.05d	5.84±0.07d	^†^
3^rd^ nymphal duration	^†^	31.00±0.19a	18.09±0.21b	15.85±0.10b	9.68±0.05c	6.07±0.07d	6.04±0.04d	6.24±0.07d	^†^
4^th^ nymphal duration	^†^	^†^	20.44±0.17a	16.83±0.08b	9.59±0.07c	6.27±0.04d	6.08±0.04d	6.48±0.05d	^†^
5^th^ nymphal duration	^†^	^†^	20.78±0.22a	17.38±0.07b	9.86±0.07c	6.55±0.05d	5.94±0.06d	6.72±0.05d	^†^
6^th^ nymphal duration	^†^	^†^	20.72±0.24a	17.89±0.07b	10.17±0.08c	6.52±0.06d	6.08±0.08d	6.89±0.05d	^†^
7^th^ nymphal duration	^†^	^†^	21.04±0.18a	18.20±0.07d	10.84±0.08c	7.74±0.06d	7.43±0.08d	7.27±0.11d	^†^
8^th^ nymphal duration	^†^	^†^	20.63±0.28a	18.52±0.07b	12.93±0.09c	9.01±0.10d	9.49±0.08d	10.97±0.18d	^†^
9^th^ nymphal duration	^†^	^†^	21.00±0.21	18.82±0.10	ND	ND	ND	ND	^†^
10^th^ nymphal duration	^†^	^†^	21.75±0.31	ND	ND	ND	ND	ND	^†^
Pre-adult duration	^†^	^†^	20.36±0.03a	19.06±0.09a	14.12±0.09b	3.35±0.11c	2.82±0.11d	3.85±0.15d	^†^
Egg-adult female	^†^	^†^	254.23±4.07a	196.16±0.60b	116.87±0.65c	66.80±0.29d	65.88±0.34d	68.50±1.54d	^†^
Egg-adult male	^†^	^†^	256.46±4.62a	206.26±0.89b	115.85±0.77c	66.87±0.27d	63.75±0.58d	66.17±1.54d	^†^

Means in the same row followed by the same letters are not significantly different (Student-Newman-Keul’s test: P<0.05).

ND–not detected (i.e. no further molting detected)

^†^- failure to develop to next instar.

**Fig 1:**
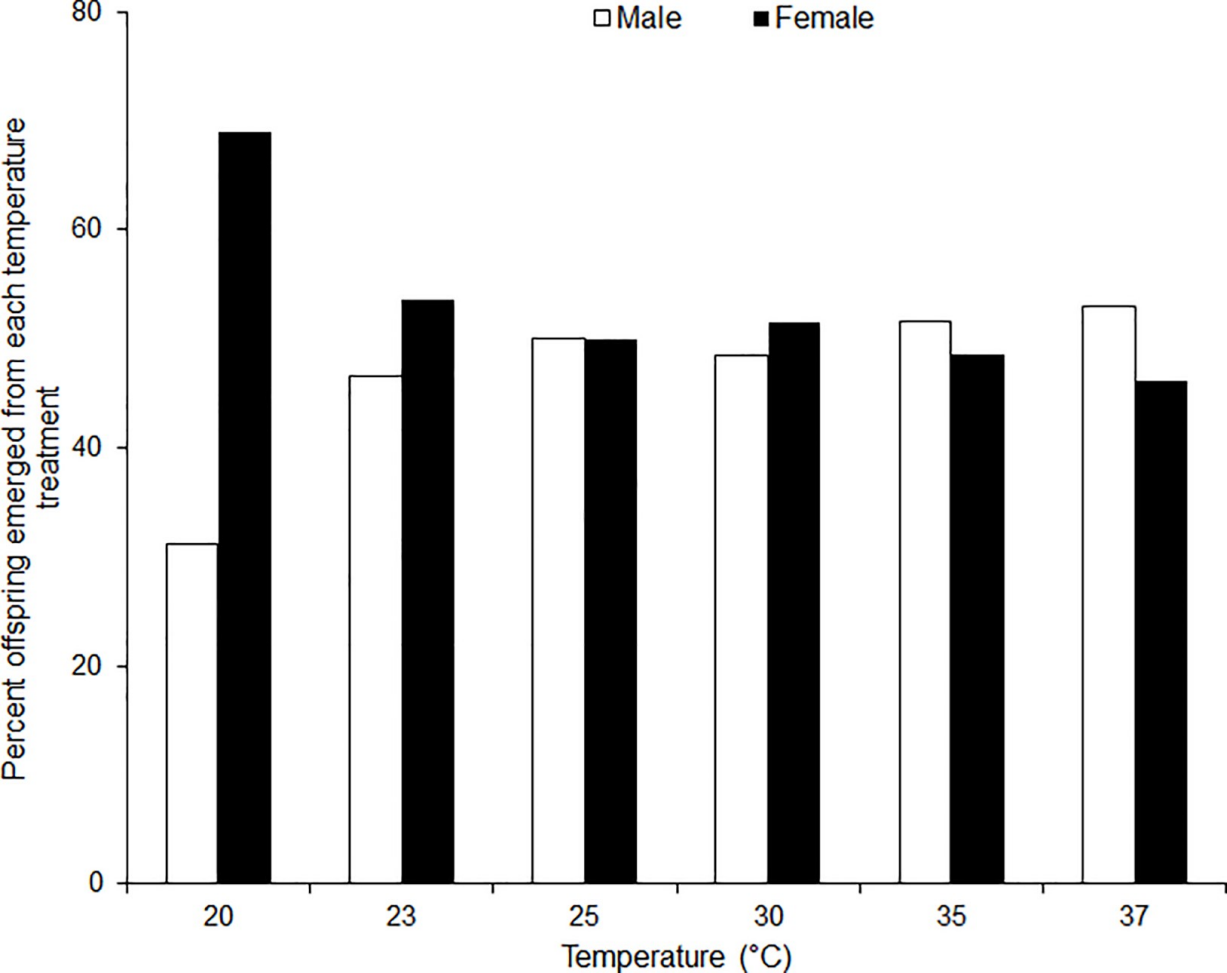
Sex ratio of *Scapsipedus icipe* adults that emerged from the last instar nymph reared at different constant temperature regimes.

Average developmental time of the immature life stages of *S*. *icipe* varied considerably (eggs: F_5, 5226_ = 11371, P < 0.0001; nymphs: F_5, 214_ = 91.67, P < 0.0001; Pre-adult: F_5, 408_ = 2065, P < 0.0001) across the various temperature treatments (Table 1). The developmental time from egg to adult varied significantly across the different temperatures (Female: F_5, 208_ = 2447, P < 0.001; Male: F_5, 193_ = 4533, P < 0.001). The average developmental time of the eggs decreased from 76.2 days at 15°C to 9.6 days at 35°C. Similarly, the mean developmental time of the nymphal stages was significantly (F_29, 340_ = 695.6, P < 0.001) shorter (3.9 times) at 37°C compared to that recorded at 20°C (Table 1). The developmental rate of each immature life stage of *S*. *icipe* was best described by the Logan 1 function and Allahyari function (Fig 1; Table 2 and Table 3). Interestingly, the sex ratio of *S*. *icipe* was female-biased at 20, 23 and 30°C, while that at 35 and 37°C was male-biased (Fig 1). For adult longevity, male crickets lived significantly longer than the females when subjected to various temperatures regimes (F_5, 401_ = 7.60; P < 0.001). The same trend was observed for the total lifespan of the crickets (F_5, 401_ = 9.65; P < 0.001) when exposed to the different temperature treatments.

**Table 2:** Estimated parameters of linear and Logan model for effect of temperature on developmental rate (1/day) for egg stage of *Scapsipedus icipe*.

Model	Model parameters	Egg
Linear	a	-0.077±0.020
	b	0.005±0.001
	T_min_	14.33
	k	185.19
	R^2^	0.919
	
Logan 1	Y	0.002±0.001
	T_max_	39.537±0.005
	T_opt_	35.000
	p	0.199±7.375
	v	4.700±0.044
	R^2^	0.988

R^2^ ("R squared")—Coefficient of determination; T_min_ - Lower limit temperature; K—Degree-day (DD) requirements; Y, p and v: Constants values; “P” refers to the number of model parameters while “n” and “m” are constants values; T_max_ - The maximum lethal temperature; T_opt_ - Optimum temperature for survival; “a” and “b” are estimated values of the intercept and slope.

**Table 3:** Estimated parameters of linear and Allahyari model for the effect of temperature on developmental rate (1/day) for Nymph and Pre-adult stages of *Scapsipedus icipe*.

Model	Model parameters	Nymph	Pre-adult
Linear	a	-0.011±0.005	-0.470±0.163
	b	0.0009±0.0002	0.025±0.006
	T_min_	12.67	19.12
	k	1111.11	40.65
	R^2^	0.860	0.826
Allahyari	p	1.846±0.005	12.804±0.041
	T_min_	13.081±0.165	16.113±0.307
	T_max_	43.468±2.077	39.168±2.365
	T_opt_	35.000	35.000
	n	2.593±4.000	3.793±7.996
	m	0.073±0.698	0.408±76.333
	R^2^	0.964	0.950

R^2^ ("R squared")—Coefficient of determination; T_min_ - Lower limit temperature; K—Degree-day (DD) requirements; Y, p and v: Constants values; “P” refers to the number of model parameters while “n” and “m” are constants values; T_max_ - The maximum lethal temperature; T_opt_ - Optimum temperature for survival; “a” and “b” are estimated values of the intercept and slope.

### Developmental rate of *Scapsipedus icipe*

**Fig 2:**
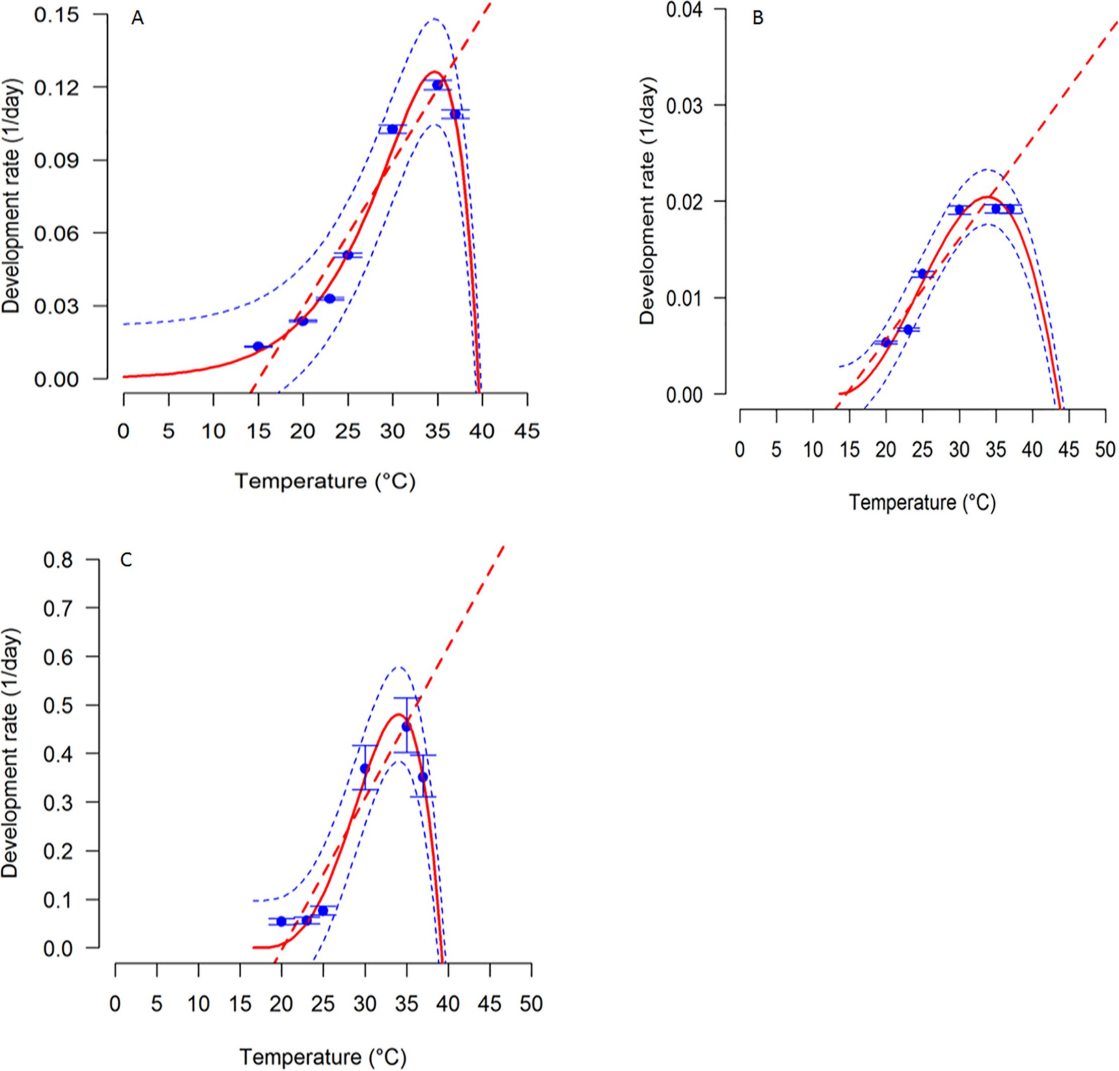
Temperature-dependent developmental rate of *Scapsipedus icipe*. (A) Egg; (B) Nymphs; (C) Pre-adult. Observed values are the solid points, with bars representing the standard deviation of the mean. Fitted models are the straight line for linear regression and a solid curved line for the Logan and Allahyari models. Dashed lines above and below represent the upper and lower 95% confidence bands.

The lowest thermal limits for development was estimated from the linear regression were: 14.3°C, 12.7°C and 19.1°C for eggs, nymphs and the pre-adult stage, respectively (Table 3). The mean thermal constant required for each life stage to complete development was 185.2, 1111.1 and 40.7, Degree days (DD) for eggs, nymph and pre-adult, respectively. The optimum temperature for development of the eggs, nymphs and pre-adult was recorded at 35°C (Fig 2). The upper-developmental threshold of *S*. *icipe* was estimated at 39.5°C for egg, 43.5°C for nymphs and 39.2°C for pre-adults (Table 3).

### Thermal effect on mortality of immature stages of *Scapsipedus icipe*

**Fig 3:**
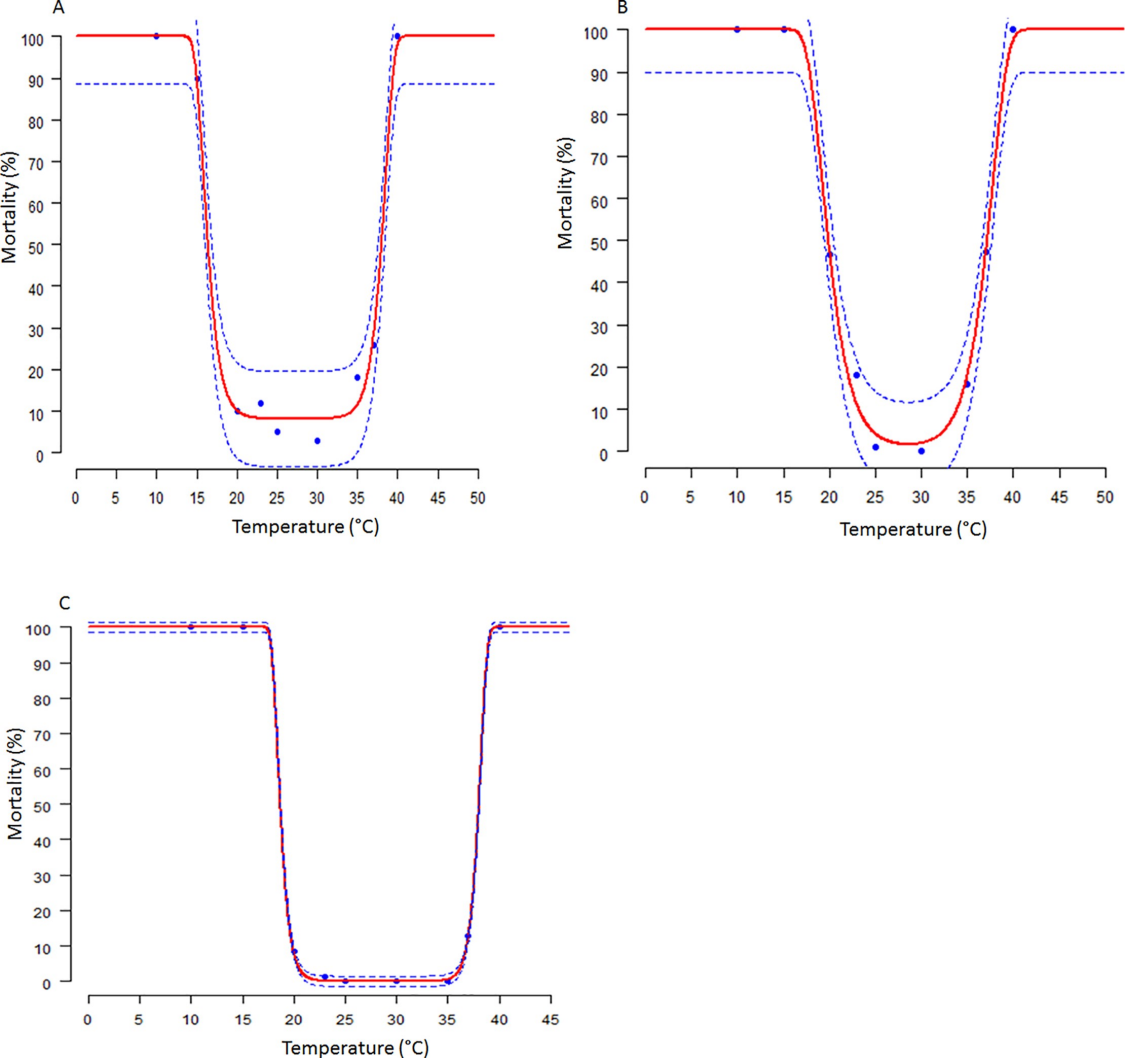
Temperature-dependent mortality rates of immature life stages of *Scapsipedus icipe*: egg (A), Nymph (B), and pre-adult (C). Fitted curves: Wang 2 model (A, B), and Wang 3 (C). Dashed lines represent the upper and lower 95% confidence.

The mortality of eggs and nymphal stages at various constant temperatures was best described by the Wang 2 function (S1 Table) while that of the pre-adult stage was best fitted by Wang 3 function (S2 Table; Fig 3).

### Thermal effect on adult body length and adult body weight

**Fig 4:**
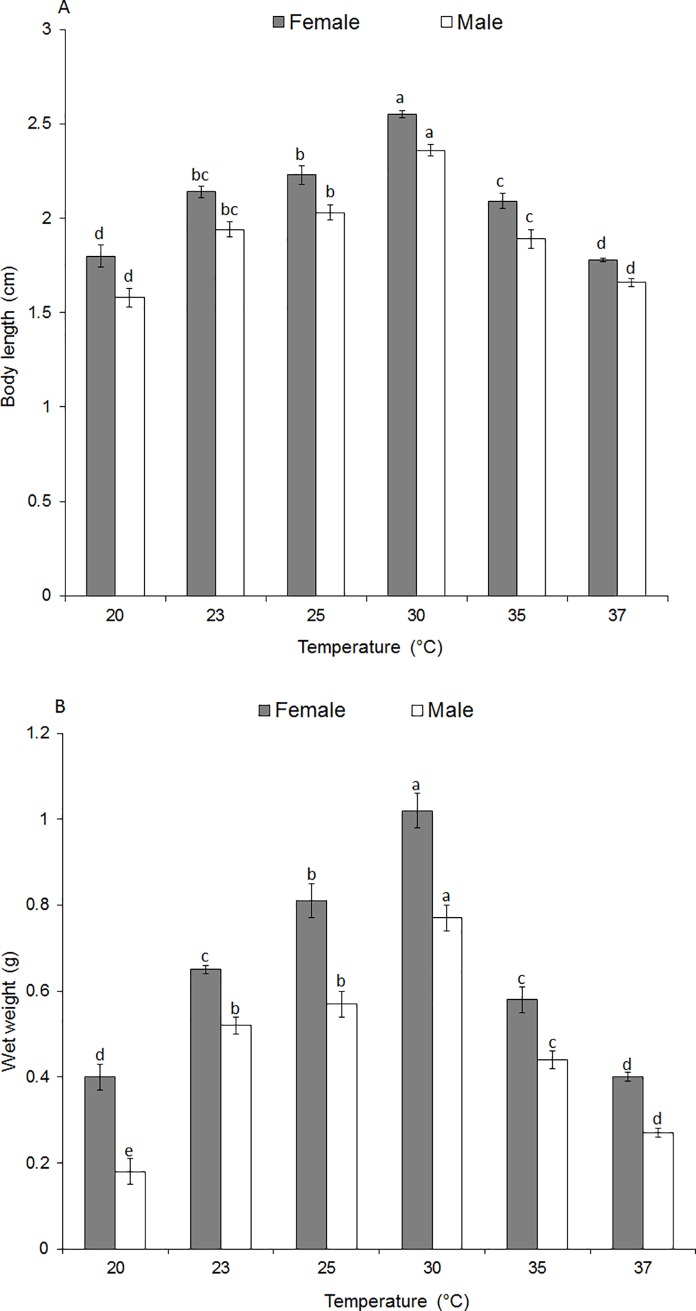
Mean (±SE) body length (A) and wet weight of *Scapsipedus icipe* females and males at six constant temperatures, respectively (B). Different letters indicate a significant difference while the same letters indicate no significant difference using Student-Newman-Keul’s test (P < 0.05).

No significant interaction was observed between *S*. *icipe*’s body length, sex and temperature (F_5,108_ = 0.39, P = 0.8500). However, there was a significant difference in male and female body length when reared at different temperatures (F_1, 108_ = 68.69, P = 0.0001; Fig 4). The highest body length of *S*. *icipe* was recorded at 30°C for both females and males, followed by 25°C and 35°C. The lowest body length was recorded at 20°C and 37°C for both sexes (Fig 4). There was a significant interaction between body weight, sex of *S*. *icipe* and temperatures (F_5, 108_ = 3.13, P<0.0001). The body weight of *S*. *icipe* males and females was found to be significantly difference when compared at different temperatures (Female F_5, 54_ = 87.22, P<0.0001; Male F = F_5, 54_ = 111.30, P<0.0001). The heaviest females and males were recorded at 30°C (Fig 4). The body length and body weight of *S*. *icipe* was found to be strongly correlated (R = 0.88; P < 0.0001).

### Thermal effect on reproduction performance of *Scapsipedus icipe*

**Fig 5:**
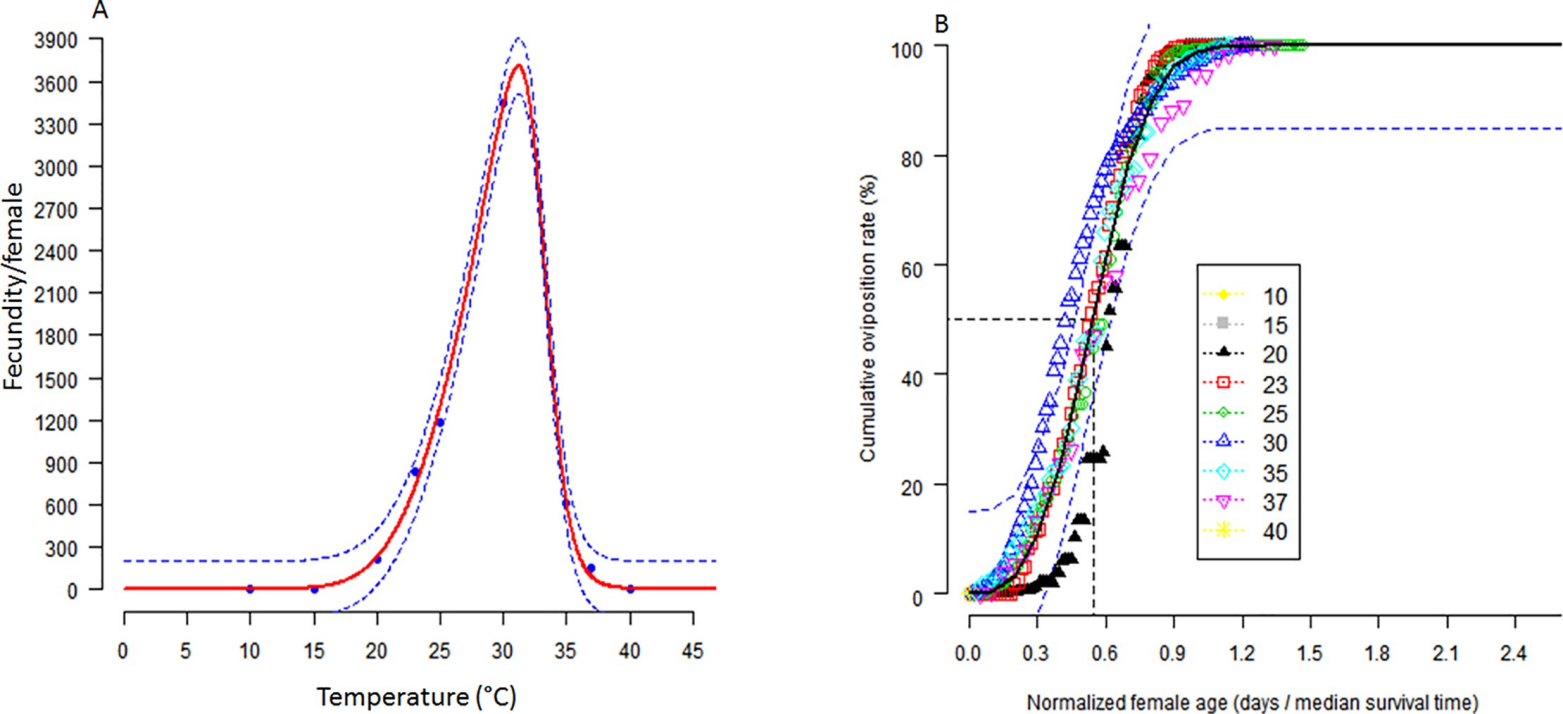
Temperature-dependent total egg production (A) and age-related cumulative proportion of egg production (B). Age of the females at 50% oviposition is indicated. Dots represent data points. The upper and lower 95% confidence intervals of the model are indicated.

*Scapsipedus icipe* was observed to lay eggs at six constant temperatures ranging between 20–37°C (Table 4). However, the total number of eggs laid per female varied considerably across the various temperature treatments (F_5, 209_ = 546.3; P < 0.0001). The effect of temperature on the overall fecundity of *S*. *icipe* was best illustrated by the Wang 7 function, which successfully predicted 30°C as the optimum temperature with the highest fecundity (Fig 5A). Lifetime fecundity of *S*. *icipe* reared at 30°C was 15.4, 4.1, 2.9, 5.6 and 21.8 times higher compared to that recorded at 20, 23, 25, 35 and 37°C, respectively (Table 4). The correlation between the cumulative portion of offspring produced per female and normalized female age was best fitted by the Exponential function (Fig 5B). The model clearly illustrated that the 60% of eggs produced by *S*. *icipe* was achieved by the time adult females reached their lifetime midpoint at 25 and 30°C. In general, 50% egg laying was completed by the time the female reached a normalized age of 0.55.

**Table 4:** The mean (±SE) of the oviposition period (days) of *Scapsipedus icipe* reared at different temperatures under laboratory conditions.

Parameter	20°C	23°C	25°C	30°C	35°C	37°C
APOP	19.84±0.88a	12.66±0.13b	8.17±0.40c	2.00±0.19d	2.33±0.18d	2.25±0.19d
Oviposition period	44.97±1.15b	45.94±0.23b	47.66±2.75b	58.82±1.95a	49.09±1.98b	16.94±0.88c
POP	11.32±0.70a	10.24±0.21a	9.43±1.21a	1.98±0.07b	2.00±013b	1.81±0.26b
Total fecundity/female	221.87±21.90e	838.24±17.23c	1182.83±52.05b	3415.98±71.27a	615.67±64.43d	156.88±19.2e

Means in the same row followed by the same letters are not significantly different (Student-Newman-Keul’s test: P>0.05). Adult pre-ovipostion period (APOP) is the period between the emergence of adult female and the onset of egg laying, Post oviposition period (POP) is the period of time from the last egg lay and when the adult female dies)

The adult preoviposition period and the total preoviposition period were inversely correlated to temperature (Table 4). Adult preoviposition (F_5, 209_ = 292.7; P < 0.001) and total preoviposition period (F_5, 209_ = 1776; P < 0.001) were significantly higher at 20°C compared to the other temperature treatments. Generally, the oviposition period (F_5, 209_ = 136.9; P < 0.001) was observed to increase drastically from 20°C to 30°C, before decreasing thereafter to reach the lowest value at 37°C. The post-oviposition period was found to varied significantly (F_5, 209_ = 41.74; P < 0.001) when compared across the different temperature treatments. The post-oviposition duration of *S*. *icipe* was significantly shorter as females died between 2–3 days, after the last egg production cycle at 30, 35 and 37°C but lived between 9–12 days at 20, 23 and 25°C (Table 4).

### Thermal effect on adult lifespan and senescence models

**Fig 6:**
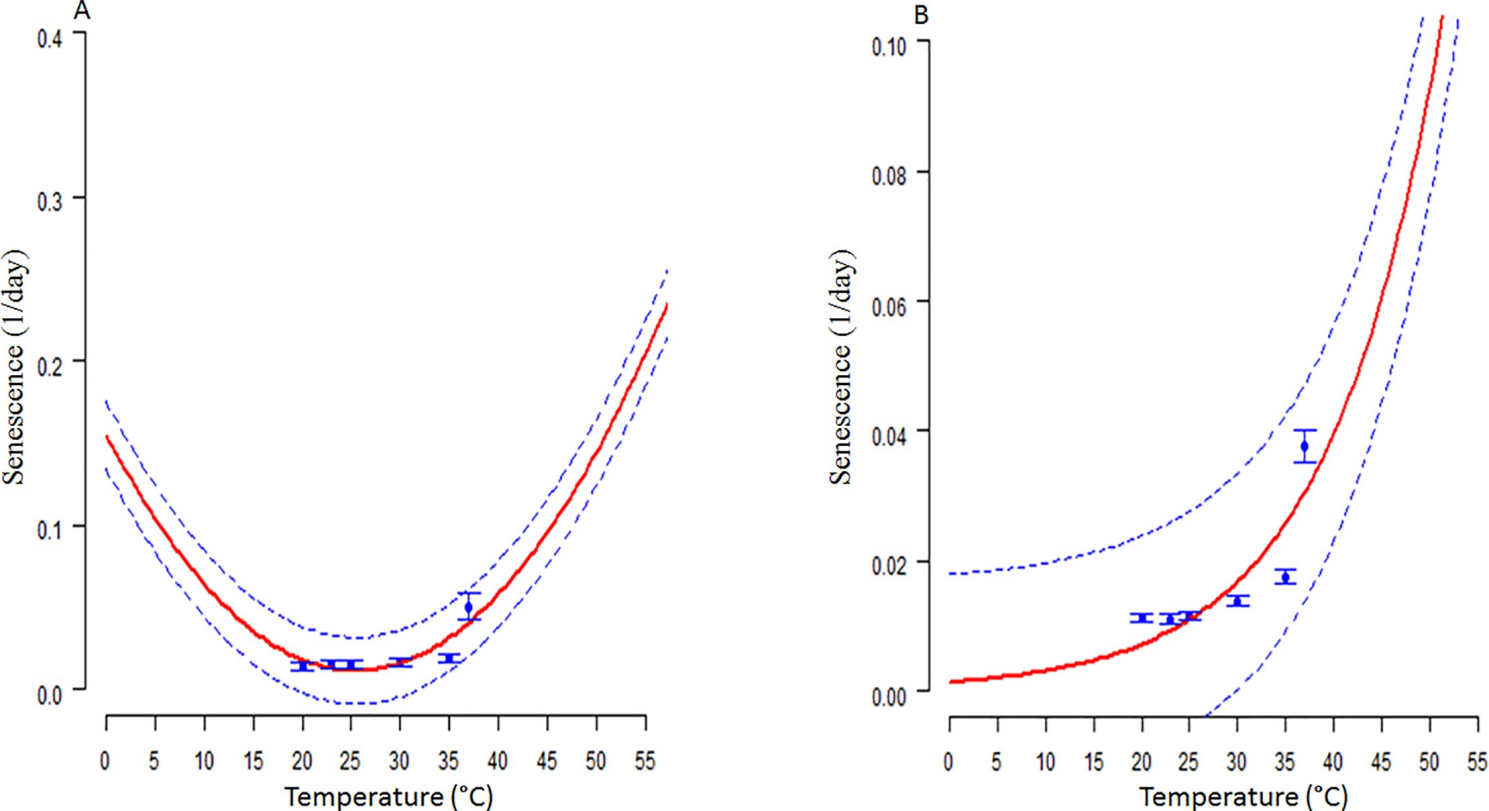
Temperature-dependent senescence rates (day 1) for *Scapsipedus icipe* adult females (A) and males (B). Fitted curves of senescence rates: Hilbert and logan 3 model (A) and Exponential simple Model (red solid line) (B). Bars represent the standard deviation of the median senescence rate.

The longevity of adult females and male *S*. *icipe* were observed to decrease significantly with increasing temperature (Table 5). The same trend was observed in the lifespan of both females and males *S*. *icipe* subjected to the same temperatures. Multiple comparisons revealed a significant difference in the longevity between adult male and female *S*. *icipe* across the various temperature regimes. The Hilbert and Logan 3 model offered a good fit to the observed mean senescence rates for adult female (S3 Table; Fig 6A) while the Exponential simple function provided a good curve for adult male (S4 Table; Fig 6B). The lowest senescence rates were observed within the temperature range of 20–25°C.

**Table 5:** The mean (±SE) of adult longevity and lifespan (days) of *Scapsipedus icipe* reared under different temperatures conditions.

Parameters	20°C	23°C	25°C	30°C	35°C	37°C
Male adult longevity	94.00±4.58a	91.06±0.25a	89.49±2.49a	72.28±1.11b	58.23±0.60c	28.44±1.67d
Female adult longevity	76.13±1.02a	68.84±0.46b	65.26±3.64b	62.80±1.97b	53.42±1.97c	21.00±0.92d
Male entire lifespan	346.00±7.66a	297.52±0.98b	204.46±2.43c	138.87±1.05d	122.65±0.76e	94.11±1.74f
Female entire lifespan	327.23±4.01a	265.08±0.77b	180.32±3.97c	129.84±2.02d	119.18±2.15e	87.44±2.29f

Data in the table are marked as mean ± SE. Means in the same row followed by the same letters are not significantly different (Student-Newman-Keul’s test: P<0.05)

### Life table parameters

**Fig 7:**
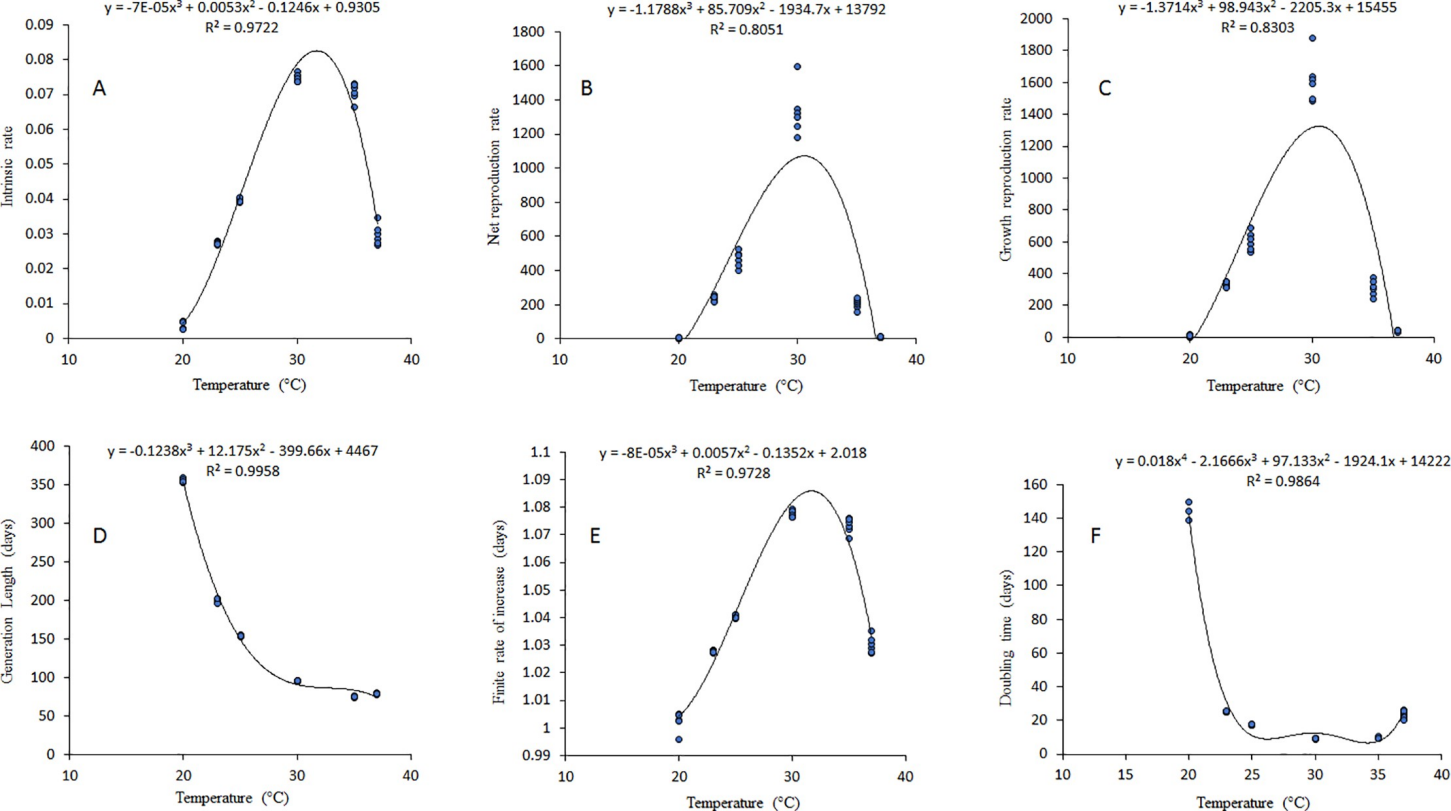
Life table parameters of *Scapsipedus icipe* estimated through model prediction over a range of six constant temperatures: [A] Intrinsic rate increase, rm; [B] net reproduction rate, Ro; [C] gross reproductive rate, GRR; [D] mean generation time, T; [E] Finite rate of increase, 𝜆 and [F] doubling time, Dt.

For *S*. *icipe* the highest intrinsic rate of increase (r_m_), net reproduction rate (R_o_), Growth reproductive rate (GRR), finite of increase (𝜆), and the shortest (Dt) was recorded at 30°C (Table 6 and Fig 7A, 7B, 7C, 7E and 7F). On the other hand, the lowest r_m_, R_o_, GRR, 𝜆, and the longest Dt were documented at 20°C (Table 6 and Fig 7A, 7B, 7C, 7E and 7F). The shortest mean generation times (T) (75.21 days) was recorded at 35°C (Table 6 and Fig 7D). Life table parameters indicate that there was a very low population growth of *S*. *icipe* at 20°C while at 30°C the population growth was significantly higher (Table 6).)The GRR was higher at 30°C with 1616.65 daughters per female compared to 12.2, 335.2, 603.5, 311.6 and 39.1 daughters per female at 20, 23, 25, 35 and 37°C, respectively (Table 6 and Fig 7C). Thus, the gross reproductive rate of *S*. *icipe* at 30°C was 132 times more than at 20°C.

**Table 6:** Simulated life table parameters of *Scapsipedus icipe* at different constant temperatures (initial egg number (n) = 100).

Temperature (°C)	Life table parameters					
	r_m_	GRR	R_o_	T (days)	𝜆	Dt (days)
20	0.004±0.001c	12.16±1.47b	4.39±0.70b	355.47±0.68a	1.00±0.001c	187.12±27.04a
23	0.027±0.001bc	335.70±6.02b	237.65±6.24b	199.420.96b	1.03±0.001bc	25.28±0.14b
25	0.040±0.001b	603.52±23.46b	466.48±18.39b	154.55±0.31b	1.04±0.001b	17.45±0.11b
30	0.075±0.002a	1616.65±57.79a	1330.76±58.37a	96.07±0.25c	1.08±0.001a	9.26±0.06b
35	0.071±0.001a	311.60±20.01b	206.16±12.34b	75.21±0.25c	1.07±0.001a	9.81±0.14b
37	0.030±0.001bc	39.12±2.29b	10.69±1.00b	79.11±0.39c	1.03±0.001bc	23.51±0.90b

r_m_: intrinsic rate of increase; GRR: gross reproduction rate; R_o_: net reproduction rate; T: mean generation time; Lambda (𝜆): Finite rate of increase; Dt: doubling time.

### Current and future climatic condition for African wide distribution of *Scapsipedus icipe*

**Fig 8:**
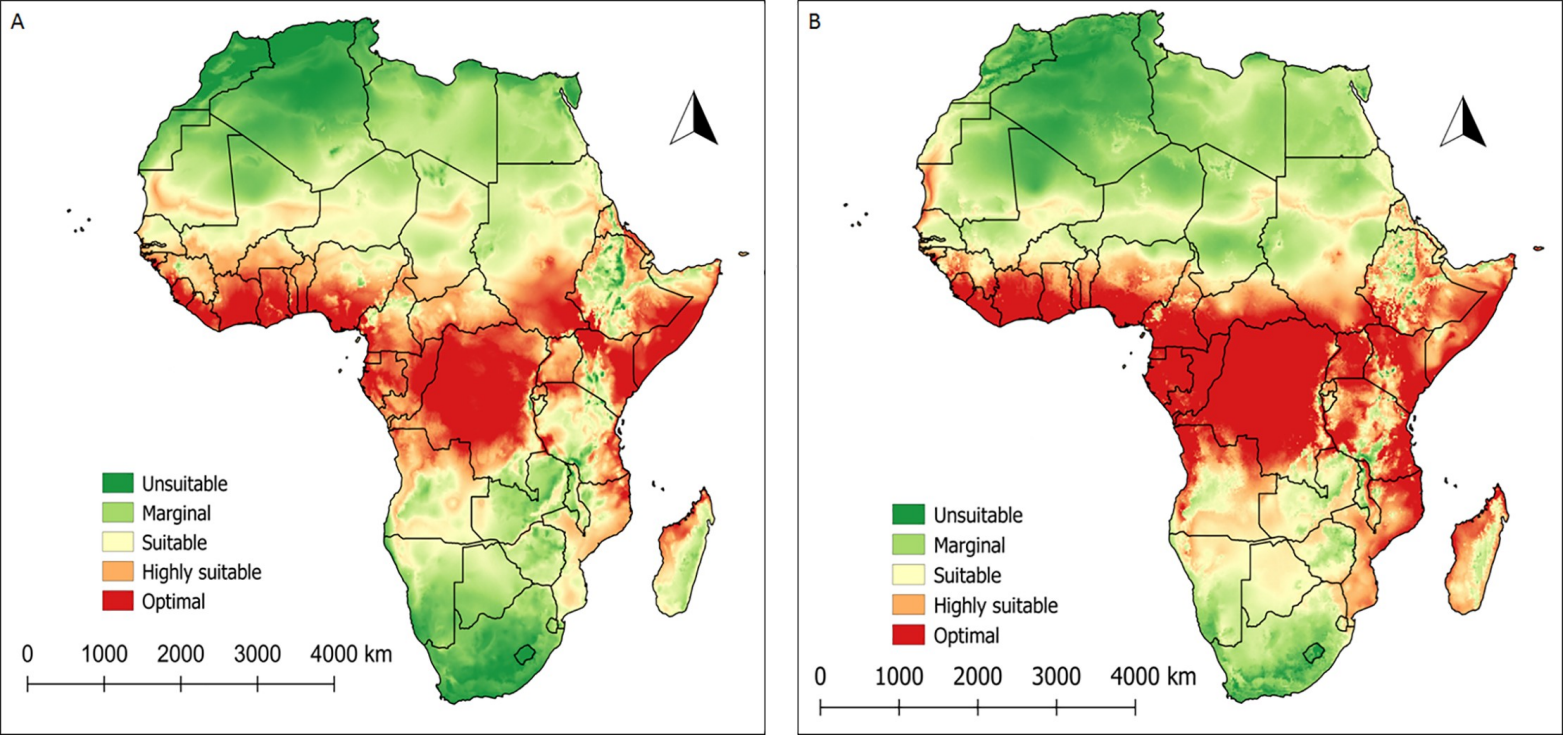
Current [A] and future [B] spatial mapping of *Scapsipedus icipe* establishment according to ILCYM model prediction in Africa.

The prediction shows that under the current climate scenario, *S*. *icipe* can establish in the tropics, especially in countries closer to the equator (Fig 8A). In Eastern Africa, the model showed that *S*. *icipe* can establish in some parts of Kenya, most of Uganda, Tanzania, Rwanda, Burundi and Southern Sudan. The potential distributional range of *S*. *icipe* also expanded to the horn of Africa (Ethiopia, Somalia, Eritrea and Djibouti). In Central Africa, the model prediction revealed that *S*. *icipe* can thrive in Cameroon, Angola, the Democratic Republic of the Congo, Equatorial Guinea, Republic of Congo, São Tomé and Príncipe. Climatic suitability of *S*. *icipe* demonstrate that this species can perform better in most Western Africa countries such as Benin, Burkina Faso, Gambia, Ghana, Guinea, Chad, Niger, Guinea-Bissau, Ivory Coast, Liberia, Mali, Mauritania, Nigeria, Senegal, Sierra Leone and Togo. For Southern Africa countries, *S*. *icipe* might be able to thrive in Mozambique and Malawi. Madagascar, which is an island in the Indian Ocean, was partly predicted to be climatically suitable. This is a promising pointer that *S*. *icipe* can be grown and utilized as food and feed in many parts of Africa because of climatic suitability (Fig 8A).

Under future climate change scenario (2050), there was a clear indication that *S*. *icipe* can expand its range to covered new elevations that were not optimal or highly suitable under the current climate scenarios (Fig 8B). For instance, in East Africa, the model revealed that *S*. *icipe* is able to establish comfortably in most of the altitudinal zones of Uganda and Kenya. In Tanzania it can establish in many areas apart from a few areas around the Central region of Tanzania. Burundi is also earmarked to be a highly suitable area where black soldier fly can thrive compared to the current climate scenarios. In Western Africa, the intensity of climate suitability in all the countries predicted was shown to be more pronounced. In southern Africa, Zambia, Zimbabwe, Botswana, Swaziland, Namibia and parts of South Africa and neighboring Zimbabwe showed marginal suitability. Madagascar was shown to be more suitability under future climate change scenario (i.e. by 2050).

## Discussion

### Effect of temperature on stage development, sex ratio, adult longevity and lifespan of *Scapsipedus icipe*

This study was aimed at providing information on *S*. *icipe* development, growth, reproduction and survival at different constant temperatures. Temperature is a known climatic factor that impacts the population growth and geographical distribution of insects [17, 49–50]. Being a new species, this is the first assessment of the effect of temperature on the life table parameters as well as its potential African-wide distribution based on temperture-dependent phenology model. The findings from our study show that temperature significantly impacts the bionomics of *S*. *icipe*. Our results concur with that of previous studies with other cricket species [51–54]. *Scapsipedus icipe* developed, survived and reproduced successfully across the various temperature regimes except at 10 and 40°C. The eggs failed to hatch 10°C, which was probably due to freezing injuries or desiccated at 40°C. At 15°C, hatched nymphs were unable to develop beyond the third nymphal stage. The number of moults to adult stage varied significantly between eight (8) and ten (10). Contrarily, other crickets such as *Gryllus bimaculatus* De Geer has 9–11 nymphal stages [55–56], *Acheta domesticus* Linnaeus has 8–15 nymphal stages [56–58] and *Acheta configuratus* (Walker) has 11 nymphal stages [56, 58]. The difference in the number of moults translates to an overall developmental time ranging between 2 and 8.5 months for *S*. *icipe* at 37°C and 20°C, respectively. Similar trend of developmental pattern has been reported for *A*. *domesticus*, which varied between 1.63 and 3.83 months when subjected at 28°C and 25°C, respectively [52]. The sex ratio was male-biased at higher temperatures (35 and 37°C) and strongly female-biased at lower temperature thresholds (20 and 23°C). However, at 25°C the sex ratio was unbiased. While a similar impact of temperature on the sex ratio has been shown earlier in the giant water bugs, *Abedus indentatus* [59] and *Belosloma flumineum* [60], our results provide the first experimental demonstration of temperature effects on the sex ratio of crickets.

### Effect of temperature on developmental rate of *Scapsipedus icipe*

Models that best describe the relationship between developmental time and change in temperature must be unimodal and estimates the lower, optimum and upper thermal requirements of the insect under study [61]. The Logan 1 and Allahyari models were the best fits properly describing the relationship between temperature and developmental rate of *S*. *icipe*. These findings are in agreement with that of previous studies by Bowling [62], who worked with *Acheta domesticus* and proof that a shift from 29.4°C to 35°C had a huge impact on the nymphal developmental duration, which decreased by 28 days. The lower development thresholds described by the linear model is in line with that for other insect species [17–18, 36, 63–65]. At the optimal temperature threshold, *S*. *icipe* had the highest survival rate accompanied by short generation time, which are considered as important criteria for mass production [65–66]. According to Patton [67], life table studies obtained under constant temperatures provide useful information on the biology and ecology of insects, which explain the dynamics that might occur within and between *S*. *icipe* populations subjected at various temperature regimes.

The oviposition duration decreased with increased and lasted for 22 days at 30°C for *S*. *icipe*, which is contrary to 9 days at 30°C for *A*. *domesticus* [67–68]. The highest number of eggs of *S*. *icipe* were laid at 30°C, while that of *A*. *domesticus* is known to occur between 27–32°C [69]. According to Adamo and Lovett [53], egg laying by *Gryllus texensis* followed that same pattern as that of *S*. *icipe*. Our results revealed that the fecundity of *S*. *icipe* increases with body size, which is in accordance to that reported for other insect species [70]. These are consistent with that of both Forrest [71] and Honěk [72], who in reference to insects, affirmed that female body size was generally a good predictor of fecundity. A positive linear or log-linear relationship between body size and fecundity (number of eggs laid over a lifespan) has been demonstrated in representatives of several insect orders [73–76]. Our results also demonstrated that *S*. *icipe* female lifespan was significantly shorter than that of the male counterparts at all temperature treatments, which agrees with that of previous studies [77–80].

### Adult body length and adult body weight of *Scapsipedus icipe*

Temperature is a crucial factor that influences the body length and body weight of crickets [52–53, 81]. Identification of the optimal developmental temperature threshold that provides bigger body size and heavy weight *S*. *icipe* in this study is a prerequisite for higher productivity, profitability and sustainable farming of the insect. Our findings revealed that the body length and body weight of female *S*. *icipe* was significantly higher compared to the males across all temperatures but both sexes experienced drastic decline of these parameters at 35 and 37°C, while optimum values were recorded at 30°C. The increase body weight and size observed at 30°C can be partially attributed to increase feed intake [53]. At each nymphal molt, body size increases by about the same factor, which leads to an exponential size increase from instar to instar, and this means that most of the body mass accumulates during the last larval instar [82]. Whether molting is gated in *S*. *icipe* by some specific factors is largely unclear, therefore more investigation is needed in this direction. However, Nijhout et al. [82] successfully demonstrated that there are developmental mechanisms that control body size and control timing events in tobacco hornworm *Manduca sexta* (L.) and the fruit fly *Drosophila melanogaster* Meigen. For examples, *Manduca* sp., has been reported to grows from about 1.2 grams to about 12 grams during its last larval instar, so growth during this instar accounts for about 90% of final body mass [82–83]. In Drosophila, the last instar larva grows from 0.5 mg to about 1.8 mg, gaining about 70% of its final mass during that stage.

### Mortality

The current study concurred with the hypotheses stated by Stevens [84], which shows that the temperature an insect can tolerate is directly proportionate to the subjected temperature change of the insect. The 100% mortality recorded at lower temperatures can be attributed to enzymatic reactions being inactivated while at higher temperatures these enzymes that play a vital role in the survival of *S*. *icipe* cricket are denatured leading to cessation life [53, 85]. The Wang 2 function [41] illustrated well the mortality of the eggs and nymphal stages of *S*. *icipe* while Wang 3 function [41] fitted best for the mortality of pre-adult stage of *S*. *icipe*. These models predicted the temperatures for optimum survival of *S*. *icipe* at 30°C, which is different from that reported by Bowling [62] for *Acheta domesticus* with optimal survival temperature at 35°C beyond which development was observed to decline.

### Life table parameters of *Scapsipedus icipe*

The life table parameters of *S*. *icipe* across the different temperature treatments varied considerably. The highest total fecundity, highest intrinsic rate of natural increase, highest net reproductive rate and shortest doubling time were recorded at 30°C. The time required for the population of *S*. *icipe* to double at temperatures ranging from 25 to 30°C varied from 17.5 days to approximately 9.3 days. Below 25°C, this time significantly increased. The regions predicted to be suitable by the model suggest that *S*. *icipe* is tolerant to a wider range of climatic conditions. This may explain to some extent the higher survival rate at the nymphal stages of *S*. *icipe* as a result of the strong endurance to each temperature conditions in our study. Reproduction remains one of the most important determinants of population fitness, especially in *S*. *icipe* that typically produce most offspring at an early age and have no parental care. Understanding the demographic parameters of *S*. *icipe* is essential to develop a profitable and sustainable mass production, given that these parameters provide the population growth rate of a species in the current and next generations [8, 86]. The present study provides novel information about life table parameters and biotic potential of *S*. *icipe*.

### Prediction of *Scapsipedus icipe* potential distribution in Africa

This marks the first attempt at modelling the distributional range of the newly described edible cricket *S*. *icipe* using temperature-dependent phenology data. The models demonstrate that *S*. *icipe* is more tolerant to a wider range of environmental conditions. The current and future scenarios remarkably align with almost all the African countries where entomophagy is widely practiced by the various communities as a traditional heritage [2]. Zooming to specific regions of the continent, our models reaffirm that Central Africa regions remains the most important biodiversity hotspot for edible insects, followed by Southern Africa, Eastern Africa and West Africa. All the areas predicted by the model have been earlier assessment by van Huis [87] and Ramos-Elorduy [88] who reported 246 species of edible insects from 27 countries and 524 species from 34 African countries, respectively. Several authors have reported that in the communities of predicted countries, it is common to find several species of insects being consume [2, 89–93]. Other countries practicing entomophagy and earmarked by the model as climatically suitable for the establishment of *S*. *icipe* included: Madagascar and Mozambique. Although the distributional range of *S*. *icipe* has not been fully documented after having been proposed as a new species, we speculate that this might not be too different from those reported by Kelemu et al. [2]. Thus, this offers plausible justification to continue to assess the occurrence of *S*. *icipe* in the African continent, to have a snap-shot of their spread to new locations. The ecological shift observed in the future scenario of the model showed that *S*. *icipe* has the potential to gradually expand its distributional range to the warmer regions of the tropics and subtropics that would favour population growth of subsequent generations of *S*. *icipe* [8, 46, 94–96].

## Conclusion

**Fig 9:**
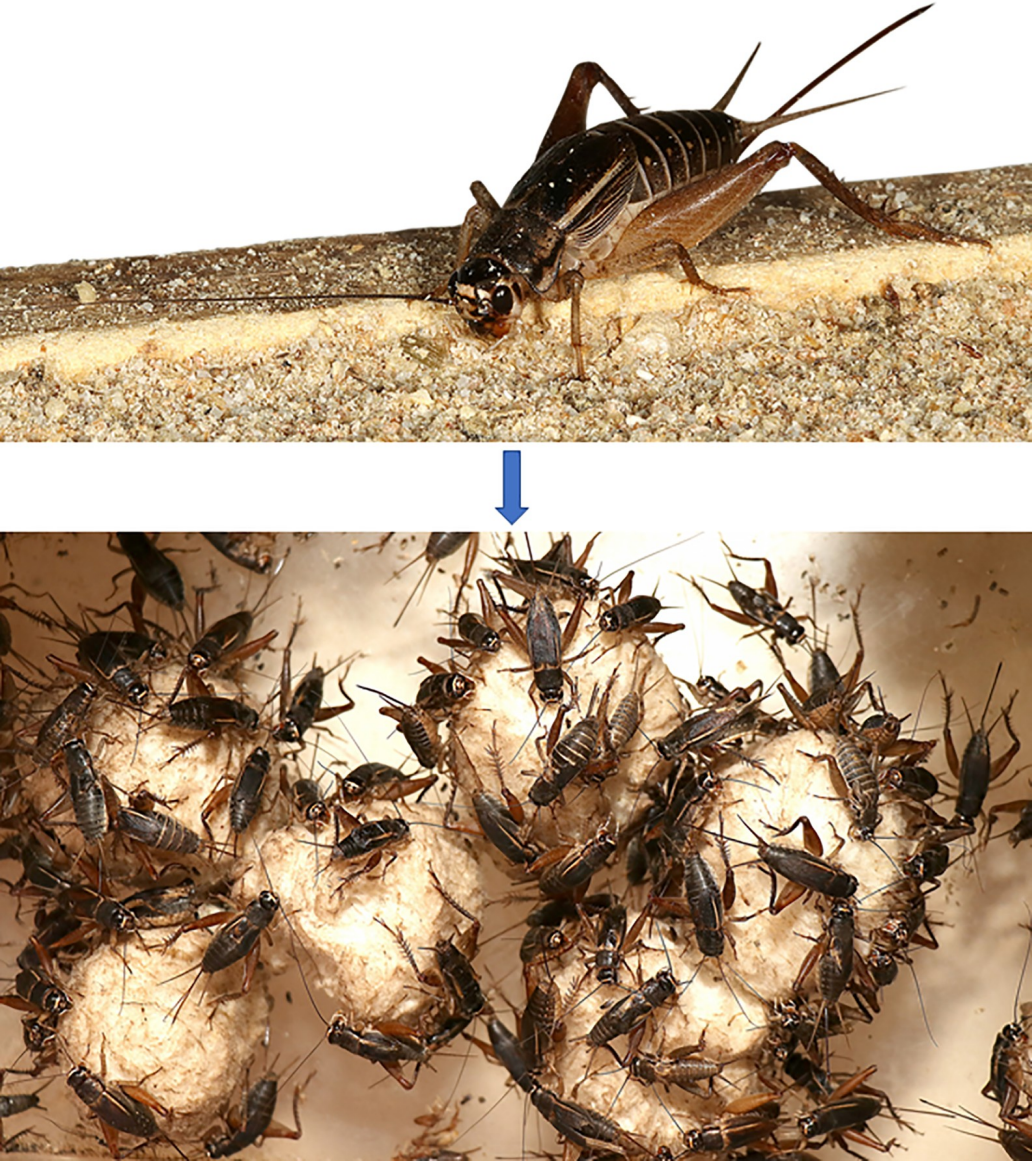
Mass production of *Scapsipedus icipe* under optimum rearing condition of 30°C (i.e. highest total fecundity (3416 individuals/female/generation), highest intrinsic rate of natural increase (0.075 days), highest net reproductive rate (1330.8 female/female/generation) and shortest doubling time (9.2 days).

We believe that the model described here is useful for optimizing mass rearing technologies for *S*. *icipe* (Fig 9) and provides addition information that may aid in decision-making. We recognize the limitations of our projected model but believe that it represents a novel technique and a potentially powerful tool for biodiversity conservation and further research on *S*. *icipe* and other edible insects as food and feed in Africa and beyond.

## Supporting information

S1 Table Estimated parameters of the Wang 2 model fitted to the temperature-dependent mortality rate for egg and nymph life stages of *Scapsipedus icipe*. (DOCX)

S2 Table Estimated parameters of the Wang 3 model fitted to the temperature-dependent mortality rate for the Pre-adult life stage of *Scapsipedus icipe*. (DOCX)

S3 Table Estimated parameters of the Hilbert and Logan 3 model fitted to the temperature-dependent senescence rate for female life stage of *Scapsipedus icipe*. (DOCX)

S4 Table Estimated parameters of the Exponential simple model fitted to the temperature-dependent senescence rate for the male life stage of *Scapsipedus icipe*. (DOCX)
