# Supplementary material for: Integrating temperature-dependent life table data into Insect Life Cycle Model for predicting the potential distribution of Scapsipedus icipe Hugel & Tanga
Source: PLoS One. 2019 Sep 25;14(9):e0222941. doi: 10.1371/journal.pone.0222941 (PMC6760797; doi:10.1371/journal.pone.0222941)
Supplement: S1 Table — (DOCX) [file pone.0222941.s001.docx]

**S1 Table:** Estimated parameters of the Wang 2 model fitted to the temperature-dependent mortality rate for egg and nymph life stages of *Scapsipedus icipe*

| **Model** | **Model parameters** | **Egg** | **Nymph** |
| --- | --- | --- | --- |
| Wang 2 | $T_{l}$ | 18.57±1.01 | 28.54±0.13 |
|  | $T_{h}$ | 35.83±0.72 | 28.54±0.13 |
|  | B | 1.09±0.24 | 1.68±0.18 |
|  | H | 0.08±0.03 | 0.004±0.002 |
|  | R^2^ | 0.9932 | 0.9948 |
|  | P | < 0.0001 | < 0.0001 |
