# Supplementary material for: Integrating temperature-dependent life table data into Insect Life Cycle Model for predicting the potential distribution of Scapsipedus icipe Hugel & Tanga
Source: PLoS One. 2019 Sep 25;14(9):e0222941. doi: 10.1371/journal.pone.0222941 (PMC6760797; doi:10.1371/journal.pone.0222941)
Supplement: S2 Table — (DOCX) [file pone.0222941.s002.docx]

**S2 Table:** Estimated parameters of the Wang 3 model fitted to the temperature-dependent mortality rate for the Pre-adult life stage of *Scapsipedus icipe*

| **Model** | **Model parameters** | **Pre-adult** |
| --- | --- | --- |
| Wang 3 | $T_{\mathrm{opt}}$ | 28.36±0.00 |
|  | B | 0.63±0.00 |
|  | H | 0.00±0.00 |
|  | R^2^ | 0.9999 |
|  | P | < 0.0001 |
