# Supplementary material for: Integrating temperature-dependent life table data into Insect Life Cycle Model for predicting the potential distribution of Scapsipedus icipe Hugel & Tanga
Source: PLoS One. 2019 Sep 25;14(9):e0222941. doi: 10.1371/journal.pone.0222941 (PMC6760797; doi:10.1371/journal.pone.0222941)
Supplement: S3 Table — (DOCX) [file pone.0222941.s003.docx]

**S3 Table:** Estimated parameters of the Hilbert and Logan 3 model fitted to the temperature-dependent senescence rate for female life stage of *Scapsipedus icipe*

| **Model** | **Model parameters** | **Female** |
| --- | --- | --- |
| Hilbert and Logan 3 | trid | 3885741.00±0.00 |
|  | $T_{\max}$ | 38.57±0.00 |
|  | $T_{\min}$ | 25.46±0.80 |
|  | D | 17554059310.00±0.00 |
|  | Dt | 0.02±0.00 |
|  | $S_{\min}$ | 0.011±0.002 |
|  | R^2^ | 0.667 |
|  | P | < 0.012 |
|  | F | 4.66 |
