# Supplementary material for: Integrating temperature-dependent life table data into Insect Life Cycle Model for predicting the potential distribution of Scapsipedus icipe Hugel & Tanga
Source: PLoS One. 2019 Sep 25;14(9):e0222941. doi: 10.1371/journal.pone.0222941 (PMC6760797; doi:10.1371/journal.pone.0222941)
Supplement: S4 Table — (DOCX) [file pone.0222941.s004.docx]

**S4 Table:** Estimated parameters of the Exponential simple model fitted to the temperature-dependent senescence rate for the male life stage of *Scapsipedus icipe*

| **Model** | **Model parameters** | **Male** |
| --- | --- | --- |
| Exponential simple | b1 | 0.0013±0.0013 |
|  | b2 | 0.0852±0.0286 |
|  | R^2^ | 0.7300 |
|  | P | < 0.03 |
|  | F | 10.80 |
